# Evaluating Partnerships to Enhance Disaster Risk Management using Multi-Criteria Analysis: An Application at the Pan-European Level

**DOI:** 10.1007/s00267-017-0959-4

**Published:** 2017-11-21

**Authors:** Stefan Hochrainer-Stigler, Anna Lorant

**Affiliations:** IIASA - International Institute for Applied Systems Analysis, Schlossplatz 1, 2361 Laxenburg, Austria

**Keywords:** Multi-sector partnerships, Multi-criteria analysis, Risk management, European Union Solidarity Fund, Insurance, Risk reduction

## Abstract

Disaster risk is increasingly recognized as a major development challenge. Recent calls emphasize the need to proactively engage in disaster risk reduction, as well as to establish new partnerships between private and public sector entities in order to decrease current and future risks. Very often such potential partnerships have to meet different objectives reflecting on the priorities of stakeholders involved. Consequently, potential partnerships need to be assessed on multiple criteria to determine weakest links and greatest threats in collaboration. This paper takes a supranational multi-sector partnership perspective, and considers possible ways to enhance disaster risk management in the European Union by better coordination between the European Union Solidarity Fund, risk reduction efforts, and insurance mechanisms. Based on flood risk estimates we employ a risk-layer approach to determine set of options for new partnerships and test them in a high-level workshop via a novel cardinal ranking based multi-criteria approach. Whilst transformative changes receive good overall scores, we also find that the incorporation of risk into budget planning is an essential condition for successful partnerships.

## Introduction

Losses from natural hazards are on the rise (UN 2015a, b). In the period of 2005–2014 average annual damages exceeded US$140 billion, which is ten times higher than three decades ago (GDFRR 2016). This trend is predominantly the result of more people and assets being located in areas exposed to natural hazards (Meyer et al. 2013). For example, the number of people exposed to river and coastal flooding globally have increased from around 520 million in 1970 to almost 1 billion in 2010 (Jongman et al. 2012). Despite these escalating losses, disaster risk management is still dominated by a “wait-and-see” approach, which is well demonstrated by the allocation of international development finances. Between 1991 and 2010 an estimated US$107 billion has been spent on natural disasters, of which 87% has gone to emergency response, reconstruction and rehabilitation and only 13% towards ex ante reduction and mitigation of disaster impacts (Kellett and Caravani 2013). Nevertheless, recent calls such as the Sendai Framework for Risk Reduction (UN 2015a, b) emphasize the need to proactively engage in disaster risk reduction, as well as to establish new partnerships between private and public sector entities in order to decrease current and future risks (see also IPCC 2012).

Very often such potential partnerships have to meet different objectives reflecting on the priorities of stakeholders involved (Reed 2008; Thompson 2013; Uhde et al. 2015; Linnerooth-Bayer et al. 2016). Consequently, potential partnerships need to be assessed on multiple criteria to determine weakest links and greatest threats in collaboration. These criteria should also include identification of missing or prerequisite elements (e.g., quantification of risk), which should be addressed beforehand to ensure the feasibility for a successful partnership (Eisenack et al. 2014). An emerging additional challenge is to combine scientific assessments and actual implementation challenges of potential partnerships to manage and reduce current and future risk within a multi-criteria setting (Scolobig et al. 2016). While there are increasing number of multi-criteria studies available at the local and community level (de Brito and Evers 2016) analysis on the supranational level are currently lacking. Our paper addresses this gap through an advanced multi-criteria approach focusing on flood risk management at the European Union (EU) level. More specifically, our study investigates the interaction between the European Union Solidarity Fund (EUSF), a Pan-European public risk pooling arrangement, insurance mechanisms and risk reduction efforts. Whilst these instruments together can efficiently address the whole risk spectrum, currently there is limited coordination between them in the EU. In order to investigate a potential multi-sector partnership, which essentially consists of the above mentioned three instruments, we develop three policy options building on quantitative risk assessment results, which are assessed against multiple criteria by applying a cardinal ranking methodology (Danielson and Ekenberg 2016). Such kind of analysis may not be only relevant at the supranational scale as demonstrated in this paper but could also be applied at lower levels.

Our paper is organized as follows. Section 2 discusses how costs and benefits of potential partnerships were determined and corresponding options based on a risk-layer approach developed. The multi-criteria approach as well as workshop setup is discussed in Section 3. Afterwards Section 4 presents the results and Section 5 discusses main conclusions. Finally, Section 6 ends with a summary and outlook to the future.

## Enhancing Flood Risk Management in the EU through Partnerships

Flooding is a prime concern not only for the EU but also for insurance providers (European Commission 2014; Zurich 2014). Our focus is therefore on current and future flood risks at the Pan-European scale and related instruments and options for partnerships to reduce them. The final result of this section is the identification of three options to be tested and evaluated in a workshop with key stakeholders using multi-criteria analysis.

### Partners and Instruments on the Pan-European Scale to Manage Flood Risk

We focus on the European Solidarity Fund (EUSF) as the main instrument at the Pan-European level. The EUSF is an ex-post loss-financing vehicle for EU member states and candidate countries, intended for use in cases in which a disaster exceeds the government’s resources to cope. Until 2014 the fund operated with an annual budget of one billion Euros. However, the latest Multiannual Financial Framework (MFF 2014–2020) has halved its budget to €500 million (2011 prices) and added a temporal risk-spreading dimension (OJ 2013). The primary aim of the EUSF is to finance emergency operations undertaken by public authorities and to alleviate non-insurable damages. Hence, it covers only a fraction of the total damages, e.g. compensation has averaged about 3% of total direct losses since 2002 (Hochrainer-Stigler et al. 2016).

Apart from the EUSF there are ongoing discussions within the EU concerning disaster risk financing, in general and disaster insurance, in particular (European Commission 2013, 2015). Experts argue that there are cases where the European NatCat insurance markets do not seem able to fully cope with existing risks (Maccaferri et al. 2012). Some of the policy discussions are thus seeking to determine how great the need is for action to enhance disaster insurance penetration at the EU level. In addition, escalating losses in the past, as well as indicators of increasing losses in the future are worrying for both public and private sector entities. Broadly speaking, discussions aim to contribute to a more disaster-resilient European Union; most importantly, they include disaster risk reduction (DRR) as an overarching aim in the field of disaster risk management (DRM). Although disaster risk management considerations have been reflected in a number of key policies, the EUSF is still the only dedicated EU-wide disaster risk financing instrument.

Important to note in this context is that in setting up the EUSF, the European Commission recognized the potential for moral hazard, in that governments may take on fewer preventive measures because they can rely on post-disaster support from the EU. Consequently, the recent reforms addressed this issue by actively encouraging member states to implement disaster prevention and risk management strategies via a requirement to report before and after applications (Hochrainer-Stigler et al. 2015). The Commission can even reduce or refuse a grant if a member state repeatedly infringes EU law regarding preventative measures (OJ 2014). The main EU legislation stipulating preventative measures for floods is the Floods Directive, which requires that countries have a risk management plan by the end of 2015 (European Commission 2014). The conditional nature of the reformed EUSF, particularly for flood risks, could send an important signal to governments to invest in disaster-proof infrastructure and other risk reducing measures. This signal will likely be strengthened as the Commission focuses on disaster risk management in response to climate change. Hence, three potential and possibly interrelated generic measures at the Pan-European level can be identified: The EUSF, insurance, and disaster risk reduction.

### Quantification of Flood Risk on the Pan-European Level

Given the selected generic measures what would be the potential mutual benefits and threats of these in monetary terms on the Pan-European scale? To answer this question a risk based assessment of potential flood losses needs to be performed. To the authors’ best knowledge the only model to date that provides risk-based information on this scale is the one by Jongman et al. (2014) and it is therefore employed for our analysis here. Table 1 below summarizes some key outcomes of the aforementioned assessment for the business-as-usual scenario (BAU). The table differentiates between insured losses (insurance claims), uninsured losses, and EUSF payments. For example, average annual flood losses are expected to increase from the current level of €4.9 billion to €23.5 billion by 2050 (the sum of those three categories). The average annual uninsured losses are expected to increase to €17.55 billion by 2050, while at the same time insurance claims could more than double from €1.89 billion to €4.64 billion. More interestingly, average annual payments from the EUSF could increase from the current level of €350 million to €1.29 billion. As a reminder, the annual budget of the Fund has been recently reduced from €1 billion to €500 million with the possibility of using any remaining funds from the previous year and funds allocated to the following year (OJ 2013).

**Table 1:** Pan-European flood risk assessment under the ‘business as usual’ scenario. Source: as discussed in Jongman et al. (2014)

Year	Uninsured loss (billion €)	Uninsured loss (% of GDP)	Insurance claims (billion €)	Insurance sector capital requirements (billion €)	EUSF claims (billion €)	EUSF claims (% of EU budget)	Additional investment in DRR
2013	4.48	0.02	1.89	115.63	0.35	1.76	–
2050	17.55	0.04	4.64	235.94	1.29	3.18	–

Whilst the BAU scenario might be considered as an acceptable policy option by some stakeholders, one can argue that in light of increasing losses, the EU should consider to combine risk management instruments (e.g., risk reduction, insurance, and EUSF) to address the increasing risk more comprehensively. Again, Jongman et al. (2014) conducted a quantitative comparison of potential ways to lower current and future Pan-European flood risk, including an increase in insurance protection as well as enhanced risk reduction. The assessment suggests that combining various risk management instruments at the European level may result in significant benefits for the EU in terms of financial resilience against natural disasters. We therefore use these results for our suggested risk-layering approach as discussed next.

### Risk-Layering of Flood Risk Management Instruments

**Fig. 1:**
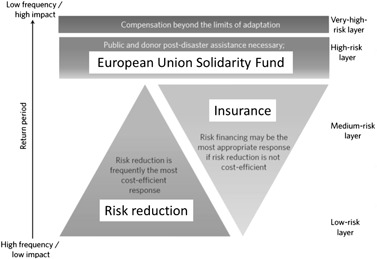
Risk layer approach. Source: Based on Mechler et al. 2014

Quantitative assessments often fail to take into account other important aspects relevant to the risk bearers involved, which raises the need for combining quantitative analysis with more nuanced qualitative approaches in order to better reflect the views and preferences of the key stakeholder groups. An additional challenge in this context is that some stakeholders may not be familiar with all risk-related concepts (Hochrainer and Mechler 2009). For the portfolio selection we employed a so-called risk-layer approach using the results by Jongman et al. (2014) (Fig. 1). The principle idea is that certain options are only preferable for certain layers of risk (e.g., in terms of probability of a flood event happening).

Figure 1 illustrates several distinct risk layers for which different adaptation options are preferable. For frequent, low impact risks, risk reduction is preferable while for more extreme layers, other instruments such as insurance or international help is needed (Mechler et al. 2014). It is important to note that risk bearers have different amounts of resources for coping with an event and therefore risk portfolios or stress thresholds (e.g., resource limits at which one is no longer able to cope with realized risks) will likely also differ. A risk-layer approach is able to discriminate between these different risk situations and is helpful for providing a clearer picture of what kind of support and risk instruments are needed for a given risk bearer. In our case, on the Pan-European level, risk reduction would be mainly used for the low risk layer, while insurance would be the main instrument for the medium risk-layer and finally, the EUSF would cover the high risk-layer. The identification of partners and instruments, the quantification of risk in combination with a risk-layer approach enables a selection of possible options to be considered within a multi-criteria approach as discussed next.

### Identification of Options for Partnerships

**Fig. 2:**
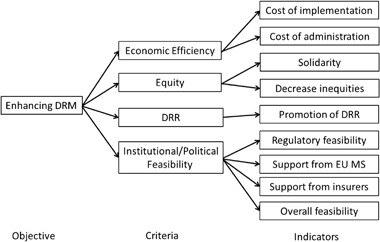
Selected criteria and indicators. Source: Adapted from Bräuninger et al. 2011

Given that the information by Jongman et al. (2014) provides probability-based estimates of losses in monetary terms, we were able to extract portfolios according to risk-layers. For example, for the low risk layer, increasing flood protection levels in all basins in Europe to a minimum of 1 per 100 years would decrease the total expected annual flood losses by around €7 billion (close to 30%) by 2050 and would cost an estimated €1.72 billion. For the middle risk layer, average modeled insured losses per year are €1.6 billion for the current period, increasing to €4.6 billion by 2050. Additionally, total flood insurance claims with a once in 200 year probability are projected to increase from €116 billion in 2013 to €236 billion in 2050. However, with risk reduction in place, insurance claims would decrease to around €81 billion today and €166 billion in 2050. Lastly, for the high risk layer, the EUSF has a risk of annual depletion of around 5 percent, with an increase to 9 percent in 2050. Current average claims are around €0.35 billion and are projected to increase to €1.29 billion by 2050. With risk reduction, average claims would decrease to €0.25 billion and €0.92 billion in 2050. Insurance could additionally help decrease ruin probabilities for the Fund. These quantitative results were complemented by a number of semi-structured (based on the criteria shown in Fig. 2) interviews with European insurance experts and practitioners (in total five experts were interviewed) surveying their insights and experience in flood risk management in general and risk pooling in particular. This led finally to the development of the following 3 policy options:

- Option 1: Eliminate the upper limit of the Fund, which is currently €500 million annually (with optional borrowing from previous/subsequent years), with the aim to respond to all qualifying disasters
- Option 2: Further strengthen the link between the EUSF and DRR. In addition to the economic performance of Member States, contributions to the Fund would also take into account risk reduction measures implemented by the country
- Option 3: Completely or partially transform the EUSF into a pre-disaster instrument that backs-up (reinsures) the national (public/private) insurance system. This would mean more affordable premiums, higher disaster insurance penetration in the EU, and less dependence on post-disaster government assistance.

In a next step, these options were tested against multiple criteria that were determined to be important for the main actors involved in these potential strategies (e.g., public sector and private sector entities, as well as non-governmental agencies and researchers). The next section discusses the multi-criteria approach adopted for evaluation of these three options.

## Multi-Criteria Analysis of Options

While the evaluation of multiple criteria by a single decision maker is rather straightforward, the comparison of many stakeholder evaluations is far from obvious and a plethora of suggestions on how this could be done can be found in the literature (Danielson and Ekenberg 2016). Recent analyzes in this field suggest that cardinal ranking methods are superior in a number of ways when compared to other ranking methods (Danielson and Ekenberg 2017). We therefore adopt this approach for our case too. In the following section, we discuss first the criteria selected and questions constructed to perform the evaluation. Afterwards, a short introduction is given on cardinal ranking methods and the specific approach applied here.

### Evaluation Criteria and MCA approach applied

The overall objective of the three options discussed above is to enhance disaster risk management through new partnerships. These options may be evaluated according to different criteria. Our criteria selection was based on work by Bräuninger et al. (2011), which developed a set of criteria and indicators to assess risk financing options for Europe very similar to our suggested ones.

As shown in Fig. 2, our four criteria are separated into economic efficiency, equity, DRR and institutional feasibility dimensions, which are all identified to be relevant to the risk bearers in our setting (but probably differ in weighting of importance, see Bräuninger et al. 2011). Economic efficiency covers the cost implications of operationalizing and running the instruments. Equity is concerned with how strongly the instrument promotes solidarity and creates inequities (winners and losers). DRR relates to the question of how much the instruments will be able to decrease risk. Last but not least, feasibility relates to the consistency of the instrument with other policy instruments and the regulatory environment, and its acceptability to the key interest groups. It should be noted that, unlike Bräuninger et al. (2011), due to the importance of DRR in our study the promotion of disaster risk reduction was introduced as a separate criterion. Considering the workshop setting, we selected a limited number of possible indicators for the questionnaire based again partly on Bräuninger et al. (2011). However, some additional questions needed to be included to allow the application of the cardinal ranking method, including the weighting of the different criteria and the set-up of questions for each option (see the options section in Supplementary A).

### Cardinal Ranking method

As indicated, for the analysis we applied a new multi-criteria (MCA) approach based on a cardinal ranking method. A review of current quantitative multi-criteria decision aid methods can be found in Danielson and Ekenberg (2016). The cardinal ranking method and its mathematical representation are discussed in detail in Danielson and Ekenberg (2017). This method was selected because it is superior to other MCA approaches in a number of ways (see again the simulation tests by Danielson and Ekenberg 2016, 2017) and could be implemented within the scope of the criteria introduced above. Furthermore, the cardinal ranking method is especially useful if weights (for us the criteria) and value functions (the related questions) can be reasonably elicited (as in our case); it preserves some comparative simplicity and the correctness found in other MCA approaches. The mathematics behind such models are surprisingly complicated and we want only to present how the weights are determined, as this is the most crucial step in any MCA approach. In our case, the cardinal weighting ranks are based on the following formula:

$$w_i^{\rm CAR} = \frac{\frac{1}{p(i)}{ + \frac{{Q + 1 - p\left( i \right)}}{Q}}}{{\mathop {\sum}\limits_{j = 1}^N {\left( {\frac{1}{p(j)}{ + \frac{{Q + 1 - p\left( j \right)}}{Q}}} \right)} }}.$$

in which *Q* represents the total number of importance scale positions and each criterion *i* has a position p(*i*) within the set *Q*. The weights can be used within value functions (e.g., showing levels of satisfaction) and therefore satisfaction levels can be made comparable across different stakeholders. What is additional important here is that apart from the methodological advantages of this method, it enables the formulation of statements rather than comparative statements. The latter can easily lead to complex computations and more importantly, to a loss of transparency on the part of the user. As indicated, we do not extend this method (for the algorithm see Danielson and Ekenberg 2016, 2017) but rather apply it to our specific case study. Therefore, the cardinal ranking method was applied via the Preference Decision Wizard, which is a recent and freely available software with an easy to handle user interface. Additionally, the tool enables the evaluation according to the cardinal ranking approach in an automated way (a detailed discussion is given in the results section). We end this section with a discussion of the workshop setup.

### Workshop Setup for the MCA

In total, 15 high level stakeholders were selected and invited for the workshop in Brussels (which was most easily to be reached for all participants). Five had a public administration background, three came from the private sector, three from not-for-profit organizations, and four from research. While the total number seems small we want to stress the fact that the participants represented carefully selected, key decision-makers in their respective fields (e.g., European Commission representatives, CEOs of insurance businesses such as Swiss Re or representative groups within the insurance business, and ministers from finance (e.g., from Romania)). In the questionnaires (see Supplementary Section A) we gave instruction to participants that they should not provide their personal views but rather, that they should evaluate the options from their professional position. While there is the open question of whether these views of such a small sample can be seen as representative, our final choice of stakeholder selection for the workshop focused on quality of representatives rather than quantity and therefore could be seen at least as indicative. Furthermore, given that we also performed qualitative guided discussions (see the discussion section), we determined that including too many participants would become problematic. The motivation of the high level stakeholders to join the workshop was incentivized due to the fact that new results (e.g., they flood risk assessment results are unique in that they present the first risk-based Pan European assessment) were presented during the meeting and there was a general interest in potential future multi-sector partnerships of the different institutions involved. The full-day workshop started off with the presentation of recent EU and member state level flood risk assessment results followed by individual presentations from key stakeholders, including representatives from the European Commission, insurance companies and public authorities from EU member states. Participants were then asked to fill out the questionnaire. The afternoon part of the workshop was dedicated to detailed plenary discussions about the feasibility, strength and weaknesses of the three policy options (see Supplementary E).

## Results

First, we present some summary statistics of the four criteria (Table 2). A 14-point Likert response scale was used to assess each question to allow for sufficient variability. Regarding economic efficiency, the mean response was around 9.93 and the median was 10, respectively. The minimum weight for this criterion was 5 while the highest weight was 14. The average score for the equity criterion was similar to that of the economic efficiency criterion, with a mean around 9.9 and a median of 9, respectively. Promotion of DRR and institutional feasibility received higher mean scores than the economic efficiency criterion. Interestingly, institutional feasibility received the highest average score, with a mean of 11.13 and median of 12. When looking at the range of scores, the minimum score for the promotion of DRR and for equity was 7. In comparison, 4 and 5 were the lowest scores for institutional feasibility and economic efficiency, respectively. An indication (also when looking at the deviation and skewness) that stakeholders had divergent views about the weights for these criteria.

**Table 2:** Summary statistics for the criteria weights

	Weighting criteria			
	Economic efficiency	Equity	Promotion for DRR	Inst./ political feasibility
Mean	9.9	9.9	11.1	11.1
Median	10.0	9.0	11.0	12.0
SD	2.6	2.4	2.6	2.9
Skewness	−.06	.3	−.3	−1.3
Range	9	7	7	10
Minimum	5	7	7	4
Maximum	14	14	14	14

No significant correlations between the criteria were found; however, the weighting of the criteria differed between stakeholder groups. For example, among the private sector stakeholders, the mean and median were highest for economic efficiency, at 12.67 and 13, respectively. For the public administration group, equity was the most important criterion with a mean and median of 10.8 and 11.0, respectively. Interestingly, among the researchers, institutional feasibility was the highest scoring criterion with a mean of 12.75 and median of 13.00. Risk reduction received the highest mean score from both the private sector and the not-for-profit groups. It should be noted that direct comparisons, as done here, are only indications and have to be treated with caution. The individual weights may not reflect the same magnitude of importance when compared to other individuals.

The different indicators and corresponding scores are provided in a summary table in Supplementary B and only the main observations are discussed next. Regarding the regulatory indicator, the enhanced link to DRR option showed the highest score. The public administration stakeholders saw the DRR option as the most feasible option, as did the private sector stakeholders. This view was shared by the not-for-profit background stakeholders and the researchers. Hence, from a regulatory point of view, for all stakeholders the enhanced link to DRR seemed to be the most promising option. For options regarding consistency with the solidarity founding principle of the EU, the uncapped EUSF option achieved the highest score overall. The uncapped EUSF was perceived to be the most consistent option with the EU solidarity principle among the public administration stakeholders. Among the private sector and the not-for-profit stakeholders option 3 was seen as the most consistent option. Among researchers, the most consistent option was the enhanced link to DRR. Summarizing, from a solidarity perspective, the public sector stakeholders thought that the uncapped EUSF was the most promising option, whereas the private sector and the not-for-profit stakeholders thought this about option 3. In contrast, the researchers provided the highest scores for the enhanced link to DRR option. While the enhanced link to DRR was seen as requiring the least additional financial resources overall, this view was not consistent across the sectors. The public administration stakeholders, the not-for-profit stakeholders, and the researcher group held this view about the enhanced link to DRR option but the private sector stakeholders saw option 3 as requiring the least additional financial resources. Administration costs were seen, overall, as being lowest for the uncapped EUSF option. This was true for all stakeholder groups except the private sector stakeholders, who saw option 3 as being the least costly. In terms of equity considerations, the scores were very similar across all three options, (e.g., around 6 out of a possible total of 14 points). The public administration stakeholders assume larger equity issues in regards to the DRR enhancement while the private sector group sees this more for the transformation option, which is the same for the not-for-profit and research groups. In more detail, for the public administration group the lowest support is for the uncapped EUSF and the highest for DRR, for the private sector group the lowest support is for the uncapped EUSF and the highest for DRR and this is in line for the not-for-profit and research groups too. Hence, enhancing DRR is seen as mainly supported by EU member states while the uncapped EUSF is seen to be experiencing strong opposition. In regard to the perception and support or opposition from an insurance perspective, enhancement in DRR is getting most support and uncapping the EUSF the strongest opposition. This trend was seen across all sector stakeholders. The most feasible option was the enhanced link to the DRR option, and the least feasible was the uncapped EUSF. Again, this trend was the same for all sub-groups within the sample. It should be noted here that option 3 was always preferable to the uncapped EUSF option. Finally, the enhanced link to DRR was also seen to be the best way to incentivize DRR and, surprisingly, the best option across all the sub-groups. In a next step, we now combined the indicator scores with the weighting of the evaluation dimensions using the cardinal ranking method.

**Fig. 3:**
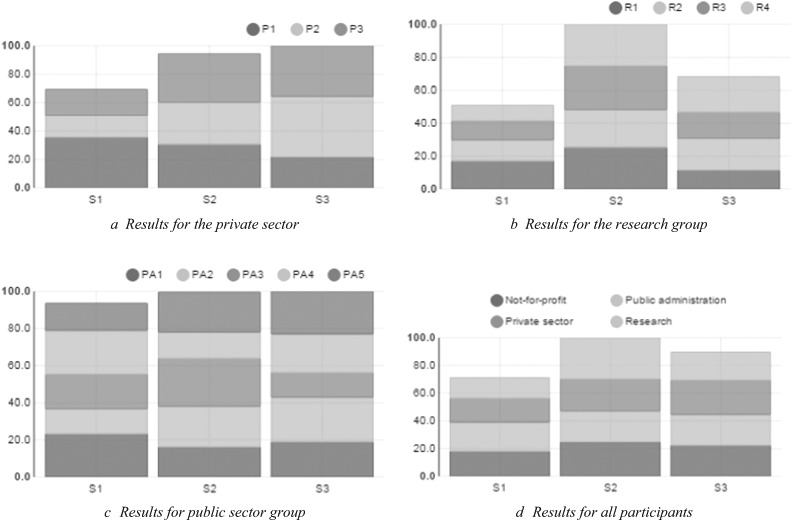
**a**–**d** Results for the different and all groups. S1, S2, S3 are referring to options 1, 2 and 3. P1, P2,… refer to the specific stakeholders within Fig. (**a**, **b**, **c**). Figure **d** is showing the results in terms of the 4 stakeholder groups

As discussed, one cannot assume that weights and indicators are valued in the same way by all decision makers. Therefore, the cardinal ranking method was applied via the preference decision wizard. Figure 1 in the Supplementary Section C shows the full MCA stakeholder tree used for the analysis, starting from the whole sample group (left hand side) and spanning up to the different stakeholder groups, the individual stakeholders in each group, and the indicators (which are weighted by the criteria weights). Due to the possibility to link the specific results to the stakeholders involved in the workshop we have to neglect the full presentation of each stakeholder perception on the options introduced and focus rather on aggregated results (a single stakeholder group example and discussion of results can be found in Supplementary D). Figure 3a shows the outcome of the analysis of all stakeholders in the private sector group using bar graphs where the *y*-axis can be interpreted as satisfaction level. It shows that option 2 and option 3 were valued highly but when looking at the overall satisfaction of the group, option 3 was the preferred option. However, one could also consider the distribution of satisfaction across stakeholders, which makes option 2 the preferred option (in terms of equal distribution of satisfaction across all stakeholders).

In the research group, option 2 was the most valued overall and valued significantly higher than option 3 and 1 (Fig. 3b). It is important to highlight that option 2 did not only lead to the highest overall satisfaction scores in the research group but satisfaction is also equally distributed among stakeholders. This resulted in option 2 being superior in comparison to the other options. While the overall satisfaction levels of the three options were nearly the same within the public sector group (option 2 and 3 received the highest scores) the distribution of satisfaction for these options is worth noting (Fig. 3c). A mixed picture emerged, for example, option 2 was satisfying at approximately equal levels for all stakeholders. Option 3 seemed to be the least satisfying for stakeholder 3 but was the most satisfying for stakeholder 2. Figure 3d shows the results taking all participants weighting criteria as well as individual indicator evaluation into account. Our analysis showed that the enhancement of DRR via the EUSF (option 2) achieved the highest satisfaction levels compared to the other options. It should be noted that the most radical option, the complete transformation of the EUSF, showed overall satisfaction levels that were close to those of option 2. Moreover, all stakeholder groups showed nearly the same satisfaction levels. This is important to be considered as for policy making processes the equality of satisfaction across groups is a good starting point for going into details how such generic options could be actually implemented.

The quantitative results in combination with the key points raised during the plenary discussions (based on Fig. 2) at the workshop provided a number of interesting insights regarding a potential Pan-European multi-sector partnership. First, it was rather clear at the very beginning and nearly agreed by all participants that any strategy for possible upcoming successful partnerships have to recognize that there is no “one-size-fits-all” approach from the European to the individual member state level possible. Rather a flexible European framework would be required that allows member states to develop and implement tailor-made strategies. This is due to the fact that each country has its own ‘history’ in managing the risks which is not feasible to be changed and streamlined to a general structure. Secondly, there is a need to precisely define responsibilities in terms of disaster risk reduction and risk financing of stakeholders at different policy levels (from local to regional to national). Thirdly, prevention measures to reduce risk need to be supported in the long run and not switched away (as in the past) due to non-disaster risk related circumstances. This means that there must be a clear commitment by all participating stakeholders in the partnership that such instruments only are effective in the long run and therefore a partnership on a continued basis needs to be established. Fourthly, communication about risk financing measures and their costs and benefits is essential for understanding, valuing, and accepting multi-stakeholder partnerships. The EUSF is widely accepted, however, for a portfolio of measures clear advantages compared to single-based instruments have to be made. Fifth, DRM needs to be explicitly incorporating within the government budget and planning process. We now proceed to the discussion section which summarizes the findings.

## Discussion

The results showed quite a diverse picture of the importance of various criteria for different stakeholder groups. However, there was consensus among the stakeholders about which options may serve best for all (either in the form of overall satisfaction or of equal satisfaction levels across groups). More specifically, risk reduction in terms of further strengthen the link to the EUSF was given highest satisfaction scores, however, the complete/partial transformation of the EUSF into a pre-disaster instrument generally showed equally distributed satisfaction levels. The capped EUSF received the lowest scores and was generally seen as inappropriate for enhancing DRM. It is worth mentioning that the transformative change option three received very good scores overall. However, some conditions that should first be met were highlighted in the structured discussions. Most importantly, it was found that DRM should be explicitly included in the budget planning process, to make DRM efforts and achievements more tangible. Furthermore, this would make it possible to better communicate DRM needs as well as providing incentives to link with other instruments.

We therefore suggest that risk due to natural disasters should be explicitly incorporated into government budgets (and planning processes) in order to enable potential partnerships on this scale. This is in line with arguments made by other authors (see the discussion in IPCC 2012; Lal et al. 2012) who noted that when substantial risk of disasters is not accounted for and coupled with weak fiscal conditions, substantial additional stress may be placed on the fiscal position during extreme events (Gurenko and Zakout 2008). This may eventually lead to reduced fiscal space for public finances that fund other public investment projects. Thus to reduce fiscal vulnerability, ex ante risk management and financing measures can be taken, such as implementing risk prevention, offering state sponsored insurance to households or engaging in sovereign risk financing measures (Cummins and Mahul 2008). What is important to note is that, conceptually, this array of measures transforms the contingent disaster liability into a direct liability, which could be priced for, e.g., with certain annual premiums, fund outlays, and debt service payments. Thus, such options could help to move a part of disaster risk liabilities into regular budget practice and could lead to both improved accountability and clear incentives for risk reduction (as it would be accounted for in the budget sheets, thus promoting the implementation of such measures).

In principle this approach is not only implementable at the national level but also at the Pan-European scale. The Committee on Regional Development of the European Parliament indicated that the rationale for financing the EUSF outside of the EU budget is that it is impossible to know in advance how much will be drawn from the Fund in the course of the year (European Parliament 2012). However, this is not the case given that estimates of risk are now available. Therefore, if disasters are incorporated into the budget planning process, there is also the possibility that the benefits of risk reduction can be estimated in monetary terms (e.g., through reduction in annual average losses). As seen via the quantitative modeling approach applied at the Pan-European level, risk reduction could have many benefits also in terms of reduction in individual risks of stakeholders (e.g., an increase in robustness of the EUSF and a decrease of capital needs of insurers). If risk is explicitly budgeted for, then risk reduction investments (at least partially) could be financed by the insurance sector. Additionally, part of this decrease in risk could be transferred to decrease premiums too. Hence, a direct link between the EUSF, the government risk, and risk reduction and insurance could be made in case the risk is explicitly accounted for in the government budget and given the other pre-requisites given above (e.g., flexibility, clear responsibilities, and long term commitment) are met. The workshop showed that for all relevant stakeholder groups such an approach could be satisfactory.

## Conclusion

In this paper we have taken three risk instruments as the focal point for our assessment of possible Pan-European partnerships to enhance resilience against flood risks, namely the EUSF, insurance and risk reduction. The quantitative analysis indicated that there could be large benefits if these instruments worked together, including an increase in robustness, a decrease in overall risk, and various co-benefits. However, boundaries (whether they are institutional, political or ones relating to efficiency) need to be overcome and we suggested possible ways of moving forward to increase resilience for today and in the changing future. We suggested that one key aspect for any successful multi-stakeholder partnership for enhanced resilience of its society to catastrophic natural hazard impacts is the explicit incorporation of risk due to natural disasters within the government budget (and planning process).

On the basis of the expert judgments during the workshops and a novel multi criteria approach, we concluded that increasing the EUSF size (option 1) was the least feasible option at the moment. We also concluded that creating a stronger link between the Fund and risk reduction, or the complete transformation of the Fund (which is a more radical option) are both as accepted as good options and rated as satisfactory for many stakeholder groups. This therefore provides a way forward for thinking about how public–private partnerships could be shaped in the future. Furthermore, in order to understand comprehensively the problem faced by different key stakeholders in the management of extreme risks, incorporating different dimensions that may not be fully quantifiable and which need to be dealt with qualitatively is necessary (e.g. equity considerations). Related problems, quantitative or qualitative in nature, very often will not be possible to be solved immediately but have to be tackled in a step by step manner. Hence, an iterative risk management approach may be helpful in achieving this task as it lays out a process-based approach to deal with current and future risks (IPCC 2012). However, how such an iterative approach could be implemented within a multi stakeholder setting on such large scales needs to be further studied.

## Electronic supplementary material

Supplementary Material A

Supplementary Material B

Supplementary Material C

Supplementary Material D

Supplementary Material E
