# Supplementary material for: Evaluating Partnerships to Enhance Disaster Risk Management using Multi-Criteria Analysis: An Application at the Pan-European Level
Source: Environ Manage. 2017 Nov 21;61(1):24–33. doi: 10.1007/s00267-017-0959-4 (PMC5765198; doi:10.1007/s00267-017-0959-4)
Supplement: Supplementary file 1 — Supplementary Material A [file 267_2017_959_MOESM1_ESM.docx]

**Supplementary A: Workshop Questionnaire**

Reform options for the European Union Solidarity Fund

Please use your professional knowledge and judgement in your responses to this questionnaire. There are no right or wrong answers.

**Your background**

Please specify the area of your professional engagement.

| 🞏 | Public administration |
| --- | --- |
| 🞏 | Private sector |
| 🞏 | Not-for-profit |
| 🞏 | Research |
| 🞏 | Other, please specify |

At what scale is the main focus of your work?

| 🞏 | International/EU |
| --- | --- |
| 🞏 | National |
| 🞏 | Regional |
| 🞏  🞏 | Local  Other, please specify |

**Weighting**

Please indicate on the scale below the importance you place on the following criteria in the assessment of the European Union Solidarity Fund (EUSF), and possible future reforms.

| **Criteria** | Not important at all Very important |
| --- | --- |
| Economic efficiency | 🞏🞏🞏🞏🞏🞏🞏🞏🞏🞏🞏🞏🞏🞏 |
| Equity (advantaging the most vulnerable countries) | 🞏🞏🞏🞏🞏🞏🞏🞏🞏🞏🞏🞏🞏🞏 |
| Promotion of risk reduction | 🞏🞏🞏🞏🞏🞏🞏🞏🞏🞏🞏🞏🞏🞏 |
| Institutional/political feasibility | 🞏🞏🞏🞏🞏🞏🞏🞏🞏🞏🞏🞏🞏🞏 |

**Options**

Below we briefly describe three options that might be considered for future reform of the European Union Solidarity Fund (EUSF).Please carefully consider these options before turning to the survey questions.

**How does the EUSF work today?**

- It can be mobilized up to a maximum annual total of € 500 million (with optional borrowing from previous/subsequent years), which is raised by Member States over and above the normal EU budget
- It aims to provide financial support to countries hit by a major disaster (direct losses are either higher than EUR 3 billion or 0.6 per cent of the GNI of the country concerned), or regional disaster (direct damage exceeding 1.5% of regional GDP) or neighboring disasters (country is affected by the same major disaster as an eligible neighboring State)
- EUSF grants can be used to supplement public expenditure for emergency operations such as the provision of temporary accommodation, cleaning up, etc.
- Payouts are conditional on the implementation of EU law pertaining to disaster risk reduction (e.g. EU Floods Directive)

**Options to be considered**

Option 1 – Uncapped EUSF

- Eliminate the upper limit of the Fund, which is currently EUR 500 million annually (with optional borrowing from previous/subsequent years) with the aim to respond to all qualifying disasters

Option 2 – Enhanced link to disaster risk reduction (DRR)

- Further strengthen the link between the EUSF and DRR: in addition to the economic performance of Member States, contributions to the Fund would also take into account risk reduction measures implemented by the country

Option 3 – Formulation of multi-sector partnership (MSP) by transforming to pre-disaster reinsurance instrument

- Complete or partial transformation of the EUSF into a pre-disaster instrument that backs-up (reinsures) national (public/private) insurance systems:
  - More affordable premiums, higher disaster insurance penetration in the EU
  - Less dependence on post-disaster government assistance

**QUESTIONS**

1. Do you think that the option is feasible from a regulatory point of view?

|  | Not feasible at all | Very feasible |
| --- | --- | --- |
| Uncapped EUSF | 🞏🞏🞏🞏🞏🞏🞏🞏🞏🞏🞏🞏🞏🞏 | |
| Enhanced link to DRR | 🞏🞏🞏🞏🞏🞏🞏🞏🞏🞏🞏🞏🞏🞏 | |
| Multi-sector partnership | 🞏🞏🞏🞏🞏🞏🞏🞏🞏🞏🞏🞏🞏🞏 | |

1. Would the option be consistent with the solidarity founding principle of the EU?

|  | Not consistent at all | Fully consistent |
| --- | --- | --- |
| Uncapped EUSF | 🞏🞏🞏🞏🞏🞏🞏🞏🞏🞏🞏🞏🞏🞏 | |
| Enhanced link to DRR | 🞏🞏🞏🞏🞏🞏🞏🞏🞏🞏🞏🞏🞏🞏 | |
| Multi-sector partnership | 🞏🞏🞏🞏🞏🞏🞏🞏🞏🞏🞏🞏🞏🞏 | |

1. How much additional financial resources would the implementation of the option require from EU Member States?

|  | Much | Few |
| --- | --- | --- |
| Uncapped EUSF | 🞏🞏🞏🞏🞏🞏🞏🞏🞏🞏🞏🞏🞏🞏 | |
| Enhanced link to DRR | 🞏🞏🞏🞏🞏🞏🞏🞏🞏🞏🞏🞏🞏🞏 | |
| Multi-sector partnership | 🞏🞏🞏🞏🞏🞏🞏🞏🞏🞏🞏🞏🞏🞏 | |

1. How costly would it be to administer the option?

|  | Expensive | Cheap |
| --- | --- | --- |
| Uncapped EUSF | 🞏🞏🞏🞏🞏🞏🞏🞏🞏🞏🞏🞏🞏🞏 | |
| Enhanced link to DRR | 🞏🞏🞏🞏🞏🞏🞏🞏🞏🞏🞏🞏🞏🞏 | |
| Multi-sector partnership | 🞏🞏🞏🞏🞏🞏🞏🞏🞏🞏🞏🞏🞏🞏 | |

1. Will the option lead to inequities (winners and losers) across Member States?

|  | Very much | Not at all |
| --- | --- | --- |
| Uncapped EUSF | 🞏🞏🞏🞏🞏🞏🞏🞏🞏🞏🞏🞏🞏🞏 | |
| Enhanced link to DRR | 🞏🞏🞏🞏🞏🞏🞏🞏🞏🞏🞏🞏🞏🞏 | |
| Multi-sector partnership | 🞏🞏🞏🞏🞏🞏🞏🞏🞏🞏🞏🞏🞏🞏 | |

1. How likely do you think EU Member States will oppose or support the option?

|  | Oppose | Support |
| --- | --- | --- |
| Uncapped EUSF | 🞏🞏🞏🞏🞏🞏🞏🞏🞏🞏🞏🞏🞏🞏 | |
| Enhanced link to DRR | 🞏🞏🞏🞏🞏🞏🞏🞏🞏🞏🞏🞏🞏🞏 | |
| Multi-sector partnership | 🞏🞏🞏🞏🞏🞏🞏🞏🞏🞏🞏🞏🞏🞏 | |

1. How likely do you think insurance companies will oppose or support the option?

|  | Oppose | Support |
| --- | --- | --- |
| Uncapped EUSF | 🞏🞏🞏🞏🞏🞏🞏🞏🞏🞏🞏🞏🞏🞏 | |
| Enhanced link to DRR | 🞏🞏🞏🞏🞏🞏🞏🞏🞏🞏🞏🞏🞏🞏 | |
| Multi-sector partnership | 🞏🞏🞏🞏🞏🞏🞏🞏🞏🞏🞏🞏🞏🞏 | |

1. How would you assess the overall feasibility of the option?

|  | Not feasible at all | Very feasible |
| --- | --- | --- |
| Uncapped EUSF | 🞏🞏🞏🞏🞏🞏🞏🞏🞏🞏🞏🞏🞏🞏 | |
| Enhanced link to DRR | 🞏🞏🞏🞏🞏🞏🞏🞏🞏🞏🞏🞏🞏🞏 | |
| Multi-sector partnership | 🞏🞏🞏🞏🞏🞏🞏🞏🞏🞏🞏🞏🞏🞏 | |
|  |  | |

1. Would the option incentivize disaster risk reduction?

|  | Not at all | Very much |
| --- | --- | --- |
| Uncapped EUSF | 🞏🞏🞏🞏🞏🞏🞏🞏🞏🞏🞏🞏🞏🞏 | |
| Enhanced link to DRR | 🞏🞏🞏🞏🞏🞏🞏🞏🞏🞏🞏🞏🞏🞏 | |
| Multi-sector partnership | 🞏🞏🞏🞏🞏🞏🞏🞏🞏🞏🞏🞏🞏🞏 | |
