# Supplementary material for: Evaluating Partnerships to Enhance Disaster Risk Management using Multi-Criteria Analysis: An Application at the Pan-European Level
Source: Environ Manage. 2017 Nov 21;61(1):24–33. doi: 10.1007/s00267-017-0959-4 (PMC5765198; doi:10.1007/s00267-017-0959-4)
Supplement: Supplementary file 2 — Supplementary Material B [file 267_2017_959_MOESM2_ESM.docx]

**Supplementary B: Question Scores**

|  | **Mean** | **Median** | **Std. Deviation** | **Skewness** | **Kurtosis** | **Minimum** | **Maximum** |
| --- | --- | --- | --- | --- | --- | --- | --- |
|  |  |  |  |  |  |  |  |
| **Regulatory_1** | 4.80 | 3.00 | 4.195 | 1.066 | .234 | 1 | 14 |
| **Regulatory_2** | 8.20 | 8.00 | 2.981 | .131 | -.327 | 3 | 14 |
| **Regulatory_3** | 5.73 | 5.00 | 3.218 | .515 | -.970 | 2 | 12 |
| **Solidarity_1** | 8.21 | 9.00 | 3.239 | -.763 | -.251 | 2 | 13 |
| **Solidarity_2** | 7.79 | 8.00 | 2.577 | .276 | -.079 | 4 | 13 |
| **Solidarity_3** | 6.71 | 6.50 | 4.214 | .096 | -1.314 | 1 | 13 |
| **ImplCost_1** | 2.27 | 1.00 | 2.344 | 2.893 | 9.356 | 1 | 10 |
| **ImplCost_2** | 8.13 | 9.00 | 4.068 | -.243 | -1.064 | 1 | 14 |
| **ImplCost_3** | 6.36 | 6.00 | 3.543 | .189 | -.560 | 1 | 13 |
| **AdminCost_1** | 8.73 | 8.00 | 2.987 | .186 | .228 | 3 | 14 |
| **AdminCost_2** | 6.53 | 6.00 | 2.232 | 1.039 | 1.133 | 4 | 12 |
| **AdminCost_3** | 5.79 | 6.00 | 3.286 | 1.132 | 1.985 | 2 | 14 |
| **Equity_1** | 6.14 | 5.50 | 3.739 | .189 | -1.142 | 1 | 12 |
| **Equity_2** | 5.57 | 4.00 | 3.031 | 1.427 | 1.438 | 3 | 13 |
| **Equity_3** | 6.00 | 5.00 | 2.944 | 1.019 | 2.167 | 1 | 13 |
| **Members_1** | 2.43 | 1.50 | 1.828 | 1.280 | 1.471 | 1 | 7 |
| **Members_2** | 6.50 | 6.50 | 3.006 | .347 | .794 | 1 | 13 |
| **Members_3** | 4.50 | 4.00 | 3.180 | .368 | -1.247 | 1 | 10 |
| **Insurance_1** | 4.53 | 4.00 | 3.944 | 1.176 | .858 | 1 | 14 |
| **Insurance_2** | 10.07 | 10.00 | 2.576 | -.114 | -.386 | 5 | 14 |
| **Insurance_3** | 7.40 | 9.00 | 4.657 | -.479 | -1.628 | 1 | 13 |
| **Feasibility_1** | 3.13 | 2.00 | 3.270 | 2.917 | 9.743 | 1 | 14 |
| **Feasibility_2** | 8.33 | 9.00 | 2.944 | -.552 | -.197 | 3 | 13 |
| **Feasibility_2** | 4.93 | 5.00 | 3.494 | .841 | .623 | 1 | 13 |
| **RReduction_1** | 4.07 | 3.00 | 3.693 | 1.009 | -.687 | 1 | 11 |
| **RReduction_2** | 10.33 | 11.00 | 2.469 | -1.297 | 2.081 | 4 | 14 |
| **RReduction_3** | 7.21 | 7.00 | 3.806 | -.270 | -.754 | 1 | 13 |

Summary statistics of question scores: Questions separated according to the three options and in the same order as in the questionnaire, see Supplementary A. E.g. Regulatory_1 is related to the regulatory feasibility question (1) and results presented for option 1, the uncapped EUSF.
