# Supplementary material for: Evaluating Partnerships to Enhance Disaster Risk Management using Multi-Criteria Analysis: An Application at the Pan-European Level
Source: Environ Manage. 2017 Nov 21;61(1):24–33. doi: 10.1007/s00267-017-0959-4 (PMC5765198; doi:10.1007/s00267-017-0959-4)
Supplement: Supplementary file 3 — Supplementary Material C [file 267_2017_959_MOESM3_ESM.docx]

**Supplementary C: MCA Stakeholder Tree**


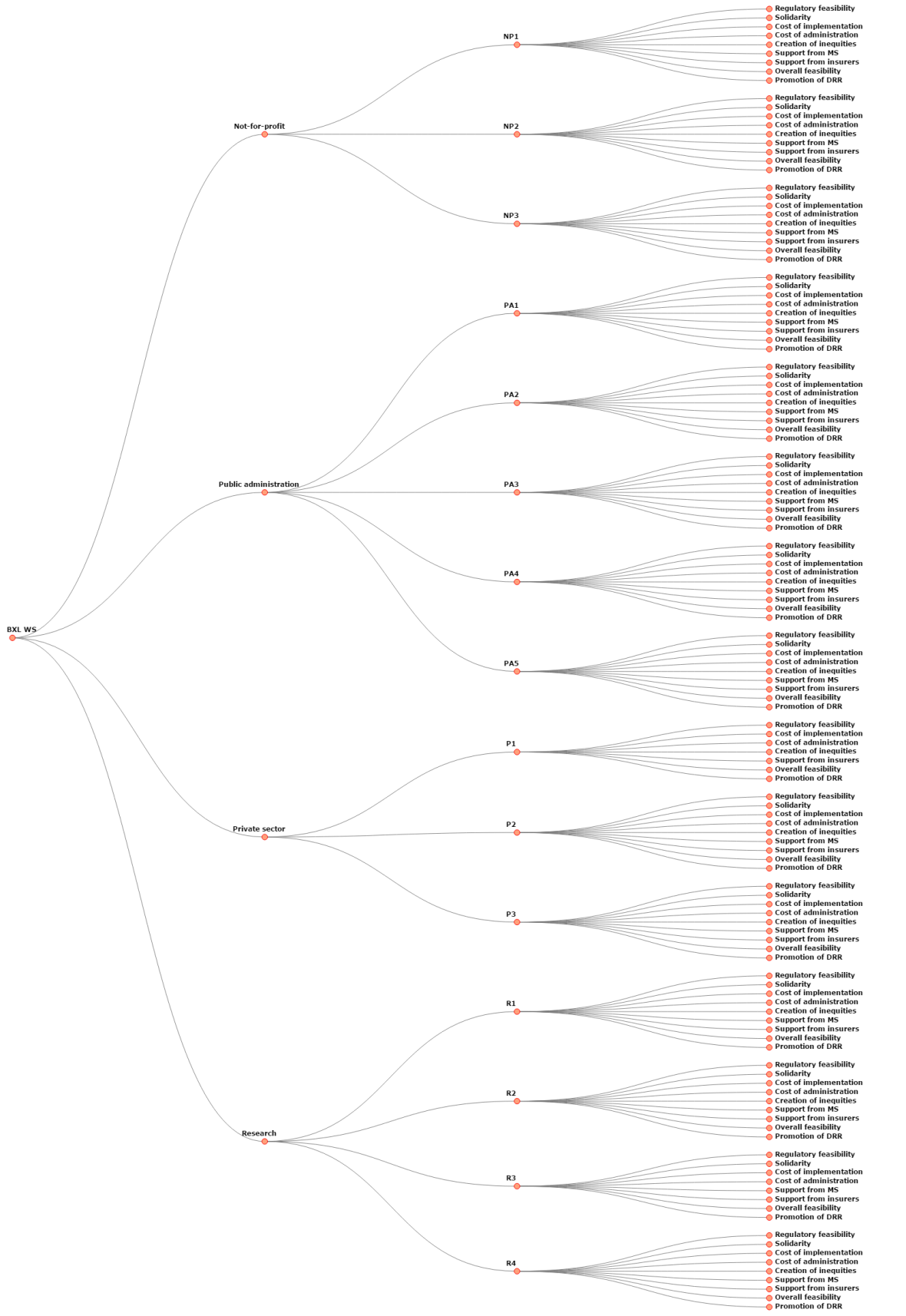


| **All** | **Stakeholder Groups** | **Stakeholders (abbreviated)** | **Questions Asked** |
| --- | --- | --- | --- |
