# Supplementary material for: Evaluating Partnerships to Enhance Disaster Risk Management using Multi-Criteria Analysis: An Application at the Pan-European Level
Source: Environ Manage. 2017 Nov 21;61(1):24–33. doi: 10.1007/s00267-017-0959-4 (PMC5765198; doi:10.1007/s00267-017-0959-4)
Supplement: Supplementary file 4 — Supplementary Material D [file 267_2017_959_MOESM4_ESM.docx]

# **Supplementary D: Stakeholder Group Example**

As discussed in the manuscript we present detailed results on selected stakeholders from the Not-for-profit group.


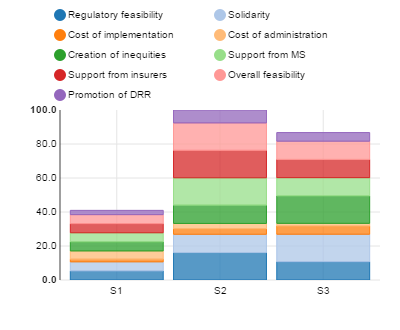

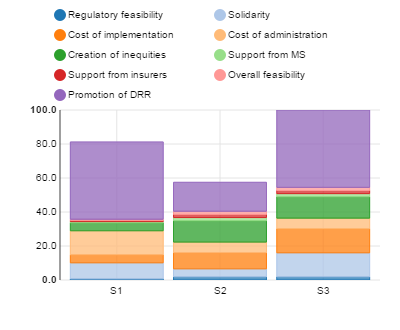


*Fig. 1 b) Results for respondent 2 in the Not-for-Profit group*

*Fig. 1 a) Results for respondent 1 in the Not-for-Profit group*


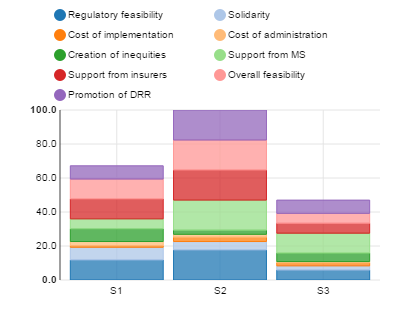

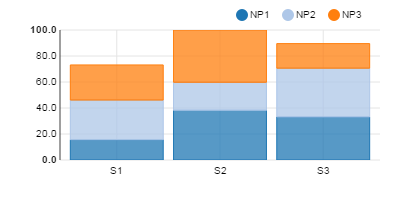


*Fig. 1 c) Results for respondent 3 in the Not-for-Profit group*

*Fig. 1d) Results for the Not-for-Profit group*

Figure 1 (a-d): Results of the MCA for the Not-for-profit sector stakeholders. S1, S2, S3 are referring to options 1, 2 and 3.

According to Figure 1a, respondent 1 showed the highest satisfaction level for option 2. Additionally, almost all single indicators for option 2 showed larger satisfaction levels compared to the other two options, with the exception of solidarity, equity and implementation costs, in which option 3 showed higher satisfaction levels. Thus one can conclude that stakeholder 1 had a higher preference (in terms of satisfaction) for option 2 and option 3, and valued option 1 the least. Stakeholder 2 (Figure 1b) valued option 3 the most, option 1 the second, and option 2 the least. Nearly all options received similar scores on most of the indicators; however, there are large discrepancies for the promotion of DRR indicator. According to stakeholder 2, this indicator showed higher satisfaction levels for option 1 and option 3. Stakeholder 3 valued option 2 (similar to stakeholder 1) the most, which was then followed by option 1 and option 3 (Figure 1c). Highest satisfaction levels are found for option 2, especially for the indicators support from MS and insurers, regulatory and overall feasibility and the promotion of DRR. The latter was seen as especially superior. Overall, option 2 was the preferred option for the not-for-profit group (Figure 1d), which is followed by option 3 and option 1. However, overall satisfaction of option 2 was achieved mainly through stakeholder 1 and stakeholder 3 and least valued by stakeholder 2. Hence, while from an overall satisfaction perspective, option 2 appears to be the most preferable, from the perspective that satisfaction should be equally distributed, option 3 may be better suited. The results can be used to address different concerns within this group, including the promotion of DRR as well as feasibility issues for the three options.
