# Supplementary material for: Evaluating Partnerships to Enhance Disaster Risk Management using Multi-Criteria Analysis: An Application at the Pan-European Level
Source: Environ Manage. 2017 Nov 21;61(1):24–33. doi: 10.1007/s00267-017-0959-4 (PMC5765198; doi:10.1007/s00267-017-0959-4)
Supplement: Supplementary file 5 — Supplementary Material E [file 267_2017_959_MOESM5_ESM.doc]

# Supplementary E: MCA Workshop Structure

# Financing natural disaster losses in the European Union – the role of insurance and the European Union Solidarity Fund

| **9:00** | Welcome  (including brief introduction to the project and its insurance working group) |
| --- | --- |
| **9:30** | State of the art approach: recent results of country and EU level flood risk assessment from 2015 – 2050 and its implications for the insurance industry and the European Union Solidarity Fund |
| **9:50** | Q&A |
| **10:00** | Flood risk in Europe: the insurance industry perspective I |
| **10:15** | Flood risk in Europe: the insurance industry perspective II |
| **10:25** | EUSF: its role, past performance and recent reforms |
| **10:40** | Disaster risk financing in the EU beyond the EUSF |
| **10:55** | Coffee break |
| **11:15** | Financing flood risk: country perspective I (Romania) |
| **11:30** | Financing flood risk: country perspective II (Austria ) |
| **11:45** | Break-out groups discussing reform options |
| **13:30** | Lunch break |
| **14:30** | Report back from the break-out groups |
| **15:15** | Short survey |
| **15:45** | Break |
| **16:00** | Discussion |
| **17:45** | Conclusions |
